# Immune exposure: how macrophages interact with the nucleus pulposus

**DOI:** 10.3389/fimmu.2023.1155746

**Published:** 2023-04-14

**Authors:** Peng Feng, Ying Che, Chunyu Gao, Liguo Zhu, Jinghua Gao, Nam V. Vo

**Affiliations:** ^1^ School of Medicine, China Academy of Chinese Medical Sciences, Beijing, China; ^2^ Department of Spine, Wangjing Hospital Affiliated to China Academy of Chinese Medical Sciences, Beijing, China; ^3^ School of Medicine, Shandong University of Traditional Chinese Medicine, Jinan, China; ^4^ Beijing Key Laboratory of Bone Setting Technology of Traditional Chinese Medicine, Wangjing Hospital Affiliated to China Academy of Chinese Medical Sciences, Beijing, China; ^5^ Ferguson Laboratory for Orthopedic and Spine Research, Department of Orthopedic Surgery, University of Pittsburgh, Pittsburgh, PA, United States

**Keywords:** nucleus pulposus, intervertebral disc degeneration, macrophage, immune exposure, MRI, histopathology

## Abstract

Intervertebral disc degeneration (IDD) is a primary contributor to low back pain. Immune cells play an extremely important role in modulating the progression of IDD by interacting with disc nucleus pulposus (NP) cells and extracellular matrix (ECM). Encased within the annulus fibrosus, healthy NP is an avascular and immune-privileged tissue that does not normally interact with macrophages. However, under pathological conditions in which neovascularization is established in the damaged disc, NP establishes extensive crosstalk with macrophages, leading to different outcomes depending on the different microenvironmental stimuli. M1 macrophages are a class of immune cells that are predominantly pro-inflammatory and promote inflammation and ECM degradation in the NP, creating a vicious cycle of matrix catabolism that drives IDD. In contrast, NP cells interacting with M2 macrophages promote disc tissue ECM remodeling and repair as M2 macrophages are primarily involved in anti-inflammatory cellular responses. Hence, depending on the crosstalk between NP and the type of immune cells (M1 vs. M2), the overall effects on IDD could be detrimental or regenerative. Drug or surgical treatment of IDD can modulate this crosstalk and hence the different treatment outcomes. This review comprehensively summarizes the interaction between macrophages and NP, aiming to highlight the important role of immunology in disc degeneration.

## 1 Introduction

Intervertebral disc degeneration (IDD) is the precursor and pathological basis of a series of spine degeneration diseases and can cause low back pain and even disability. It is also the main cause of global productivity loss. It is estimated that the prevalence of IDD-related diseases is as high as 84%, with 11−12% of the population being disabled as a result (1). The nucleus pulposus (NP) is the core structure of the intervertebral disc and plays an indispensable role in the function of the spine. The NP originates from the embryonic structures, sclerotome and notochord. Healthy NP is avascular and immunogenic as it is isolated from the immune system (2). The NP is protected from the immune environment by the annulus fibrosus, cartilage endplate, and ligament barriers (posterior/anterior longitudinal ligament) (3). Normally, these immune barriers prevent immune cells from interacting with the NP. However, from the moment of immune exposure due to disc tissue rupture, macrophages become involved in the entire process of NP degeneration, directly influencing disease prognosis, outcome, and thus clinical decision-making (4). Recent studies have indicated that the involvement of immune cells, represented by macrophages, may have a decisive impact on the function of NP cells (NPCs) (2, 5, 6). However, understanding of this process is still limited.

An abnormal immune response can lead to NP degeneration (6). Macrophages are derived from the blood mononuclear phagocytic system and prefer an eosinophilic environment. As interstitial cells, macrophages cooperate with parenchymal cells to jointly maintain tissue homeostasis (7). Conversely, they have opposite roles in immune-privileged organs, such as the NP. Under pathological conditions, infiltration of macrophages may lead to abnormal adverse consequences, e.g., degradation of normal tissues and initiation of a painful inflammatory response (8). The broad target macrophage-mediated phagocytosis removes unnecessary or unwanted tissue components (7). Macrophages are highly capable of sensing and detecting various cues and stimuli from the microenvironment (9). Thus, the NP can be monitored by the immune system, similar to the brain, where this surveillance does not take place inside but outside the tissue (10). When the NP loses its protection by the immune barriers for various reasons, it is immediately detected by the immune system, which signals the chemotaxis and aggregation of macrophages, triggering an interaction between macrophages and the NP and inducing an inflammatory cascade (2).

However, previous studies have generally focused on changes in NPCs with limited research exploring the role of the immune system in NP degeneration. Further, researchers have paid less attention to the interaction between the NP and macrophages. Herein, we summarize the entire process of immune interaction between the NP and macrophages, including the reasons for NP immune exposure, reported evidence of such interaction via histopathology and magnetic resonance imaging (MRI) studies, the macrophage-NPC crosstalk, and the role of macrophages in the treatment of IDD.

## 2 Reasons for immune exposure of the NP

**Figure 1:**
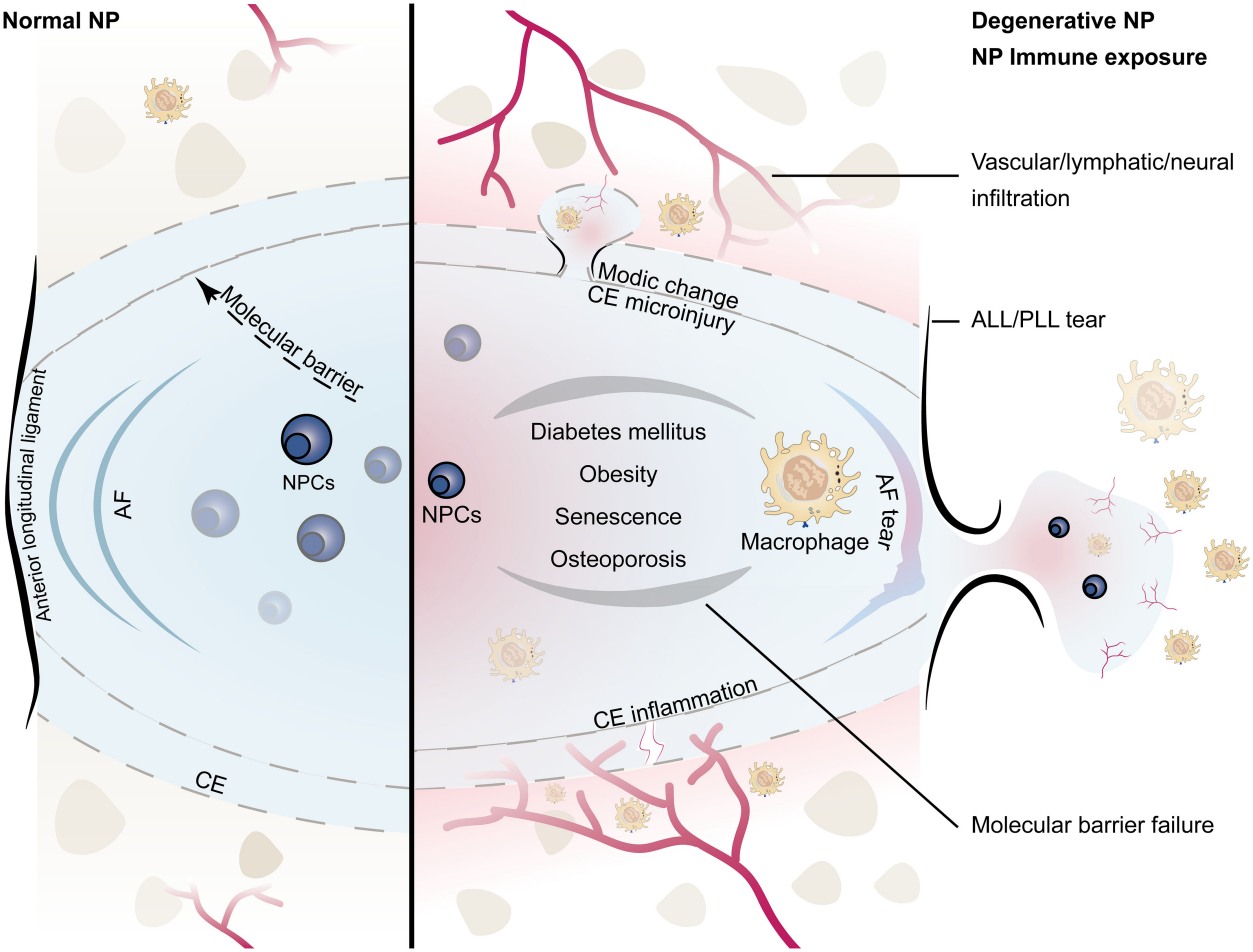
Schematic diagram of the immune barrier of the NP (left): including the AF, the upper and lower CE, the anterior and posterior longitudinal ligaments, and the molecular immune barrier; microvessels are distributed around the NP without infiltration; the height of the NP is normal. NP immune exposure and NP-macrophage interaction (right): including CE damage, AF rupture, ALL/PLL rupture, and failure of the molecular immune barrier, which are mostly related to intervertebral disc injury caused by abnormal stress; immunogenicity of the NP induces macrophage infiltration; microinjury may lead to Modic changes, while severe injury tends to induce endplate chondritis; the height of the NP decreases; the number of NPCs decreases. (NP, nucleus pulposus; NPCs, nucleus pulposus cells; CE, cartilage endplate; AF, annulus fibrosus; ALL/PLL, anterior longitudinal ligament/posterior longitudinal ligament).

A schematic diagram of the composition of the NP immune barrier and the infiltration pathways of macrophages is summarized in **Figure 1**. Immune exposure mainly occurs from disruption of the annulus fibrosus and posterior/anterior longitudinal ligament. The NP is rich in proteoglycan matrix that provides swelling osmotic pressure to counteract axial load and distribute spinal pressure evenly (11). When the local stress or shear force exceeds the bearing capacity of the intervertebral disc, the NP, owing to its incompressibility, impacts the surrounding structures, causing damage at the weakest point. Despite reinforcement of the posterior longitudinal ligament, the posterior part of the annulus fibrosus is the weakest. Since the annulus fibrosus and posterior longitudinal ligament also lack self-healing capacity, the resulting immune exposure is sustained in the short term, gradually leading to macrophage infiltration throughout the disc and triggering an inflammatory cascade (12). It is important to note that the ligament is part of the immune barrier, and if the NP only breaks through the annulus fibrosus but not the longitudinal ligament, there may be no immune exposure (4). Shigeru et al. analyzed the relationship between the posterior longitudinal ligament and inflammatory cell infiltration (especially M1 macrophages) in the NP. They found that subligamentous herniations did not show marginal enhancement on MRI and that intact ligaments prevented microvascular infiltration; further microscopic observation showed that most subligamentous herniations were free of macrophage infiltration, whereas perforated ligaments contained a large number of macrophages (4).

Immune exposure of the NP may also come from rupturing of superior/inferior cartilage endplate. There are no blood vessels and lymphatic vessels in the cartilage endplate, and the dense structure formed by the high concentration of proteoglycan also prevents the penetration of other cells (13). Once the cartilage endplate is torn or destroyed, the acidic NP content is directly exposed to the circulatory system, triggering the chemotaxis and aggregation of macrophages, resulting in inflammatory responses and disc degeneration. Johnson et al. found that high concentrations of proteoglycans in the cartilage endplate can inhibit the adhesion and migration of macrophages and endothelial cells (14). Dudli et al. found that fibrous tissue replaced normal tissue at the microscopic cartilage endplate break, and the bone-disc junction was filled with fresh granulation tissue and microvessels (15). Injury to the endplate triggers a series of degenerative changes in the intervertebral disc, which further worsen material exchange between the NP and vertebrae (16). Most cartilage endplate injuries have a clear history of trauma. The NP itself may not be damaged; however, disruption of the immune barrier forces it to be exposed to the immune system, thereby exacerbating disc degeneration.

In addition to physical barriers, molecular immune barriers also play an important role in the immune-privilege status of the NP. The molecular immune barrier is actually the process of material exchange and information transmission between the NP and the outside. Mature NP does not contain blood vessels, lymphatic vessels, and nerves, but these tissues are abundant in the periphery of the intervertebral disc, which is the basis for macrophage-NP communication (17, 18). The failure of this crosstalk leads to loss of NP microenvironment homeostasis, resulting in macrophage chemotaxis and infiltration, which indirectly promote macrophage-induced growth of blood vessels, lymphatic vessels, and nerves, ultimately leading to failure of the NP immune barrier (17, 19–21). At the same time, analyses of both human and animal samples have found that the NP contains immune-privileged molecules (such as Fas/Fasl), and these soluble proteins can act as signaling molecules to resist the infiltration of macrophages and even induce their apoptosis (19, 22). In addition, some syndromes, such as diabetes, obesity, aging, and osteoporosis, can also indirectly lead to failure of the NP immune barrier. A retrospective clinical study showed that the longer the course of diabetes, the more serious the degeneration of the NP (23). In a more in-depth experimental study, it was found that diabetes can promote endplate microvascular disease, trigger inflammation, and induce macrophages to infiltrate the NP; at the same time, a high-glucose environment affected NP metabolism and promoted the failure of the molecular immune barrier (24, 25). In obese patients, in addition to direct mechanical overload-induced intervertebral disc damage, adipocytes also participate in the NP-macrophage communication by secreting cytokines (e.g., tumor necrosis factor (TNF), interleukin (IL), leptin, adiponectin, and resistin) (26). A senescent NP has a tendency to be infiltrated by vessels, nerves, and lymphatic vessels with age. Our group also found that the aging immune system can induce NP degeneration (6). Osteoporosis also indirectly affects NP metabolism and macrophage-NP homeostasis (27–29).

These causes of immune exposure of the NP are often not isolated but synergistic. Since macrophages have different polarization types, mainly the pro-inflammatory M1 type and anti-inflammatory M2 type, these can interconvert and play different roles in tissues (30). Therefore, it is important to study in detail the interaction of macrophages with the NP when NP immune barrier is compromised.

## 3 Macrophage-NPC crosstalk

NPCs are autoimmunogenic, meaning that even non-degenerated NPCs can elicit an immune response. Subcutaneous implantation of autologous NP can trigger the infiltration of a large number of immune cells (31). An *in vitro* co-culture model of macrophages and NPCs also showed the infiltration of macrophages and a decreased wet weight of the NP (32). *In vitro* experiments showed that macrophages had a cytotoxic effect on NPCs (33). However, when NP tissues were transplanted into immunodeficient mice, it was found that the survival rate of NPCs in immunodeficient mice was significantly higher than that in wild-type mice (33). This suggests that normal NPCs can be recognized by macrophages without the protection of an immune barrier. Under pathological conditions, this crosstalk is even more intense. After the immune system recognizes the NP, macrophages first polarize toward the M1 type under the action of chemokines, triggering an inflammatory response (8). Unhealthy NPCs release more inflammatory factors, which recruit additional macrophages and ultimately cause an inflammatory cascade. As inflammatory stimuli intensify, microvessels direct infiltration, which further promotes the recruitment of macrophages (34). This interaction between M1 macrophages and NPCs may form a positive feedback system, which may lead to persistent disc degeneration and chronic pain until the autoimmunity against the NP is resolved (35). The NP targeted by autoimmunity is gradually destroyed or replaced as the degeneration grade increases; the number and type of cytokines change; and the macrophages gradually change from type M1 to M2 to carry out tissue repair work (8, 36). From a molecular regulation perspective, there is a complex network-regulation relationship between macrophages and NPCs.

### 3.1 M1 macrophage-NPC crosstalk

At present, the most common cytokine released by M1 macrophages in the NP is TNF-α; thus, researchers generally use this cytokine as an inducer to simulate the degeneration of NPCs *in vitro*. Several experimental studies across human and animal NP specimens have shown that TNF-α is highly expressed in the degenerated NP (37, 38). TNF-α is released by M1 macrophages during acute inflammation and is responsible for various intracellular signaling events leading to abnormal cell death (39). Studies have shown that TNF-α can induce the expression of SLC20A1-1 (a long non-coding RNA) in NPCs and that SLC20A1-1 is involved in TNF-α-induced NPC apoptosis by sponging miR-146a-5p (40). TNF-α released by M1 macrophages can stimulate the expression of C-C motif chemokine 2 (CCL2) and C-C motif chemokine 3 (CCL3) in NPCs and induce the release of IL-8. CCL2 and CCL3 can further activate CCR1+ and CCR2+ M1 macrophages and promote macrophage migration (41–43). TNF-α can also activate the NF-κB and p38-MAPK signaling pathways in NPCs, induce CCL4 expression, and further promote M1 macrophage infiltration (44). The released TNF-α can further activate many downstream signaling pathways in NPCs, including the NF-κB, ERK, MAPK and PI3K/AKT pathways. However, blocking TNFR1 (the TNF-α receptor) could partly inhibit the apoptosis of NPCs and expression of inflammatory factors (45, 46). NPCs induced by TNF-α undergo a series of phenotypic changes, such as mitochondrial dysfunction, oxidative stress, apoptosis, pyroptosis, and senescence (47). In addition to TNF-α, IL-1β is also an important inflammatory factor released by M1 macrophages. In a co-culture experiment of M1 macrophages and NPCs *in vitro*, M1 macrophages activated the HMGB1/Myd88/NF-κB pathway and NLRP3, resulting in increased IL-1β release (48). Kang et al. found that IL-1β can activate the IRE1 pathway, leading to the activation of the NF-κB, PI3K/Akt, and MAPK signaling pathways and endoplasmic reticulum stress in NPCs, ultimately leading to NPC apoptosis (37). Activation of M1 macrophages induces upregulation of HSP90 itself and downregulation of HSP70, which activates the JAK2-STAT3 pathway and ultimately leads to the activation of the NF-κB and MAPK pathways, stimulating the release of more inflammatory factors (49). At the same time, some researchers have explored the upstream drivers of inflammatory factors. Hasvik et al. found that in M1 macrophages, miR-17 was associated with the infiltration of macrophages and the expression of TNF-α, suggesting that it may be involved in the expression of TNF-α (50). In M1 macrophages, miR-129-5p directly targets LRG1 and promotes activation of the p38 MAPK signaling pathway, which leads to the release of inflammatory factors and maintains M1 macrophage polarization (51). The ECM of the NP is an important site for the interaction of M1 macrophages with NPCs and is inherently immunogenic. Simona et al. found direct evidence of an autoimmune response in the NP by detecting IgG against collagen types I, II, V, and aggrecan in degenerated diagnostic samples from humans, which can be recognized and infiltrated by immune cells (52). The lysosomes of M1 macrophages contain a large number of MMP enzymes. Induced by IL-1β and TNF-α, the gene expression of MMPs in macrophages is up-regulated, and their relationship with tissue inhibitor of metalloproteinases (TIMPs) is altered, which leads to ECM degradation and infiltration of tissues, such as blood vessels and nerves (53).

**Figure 2:**
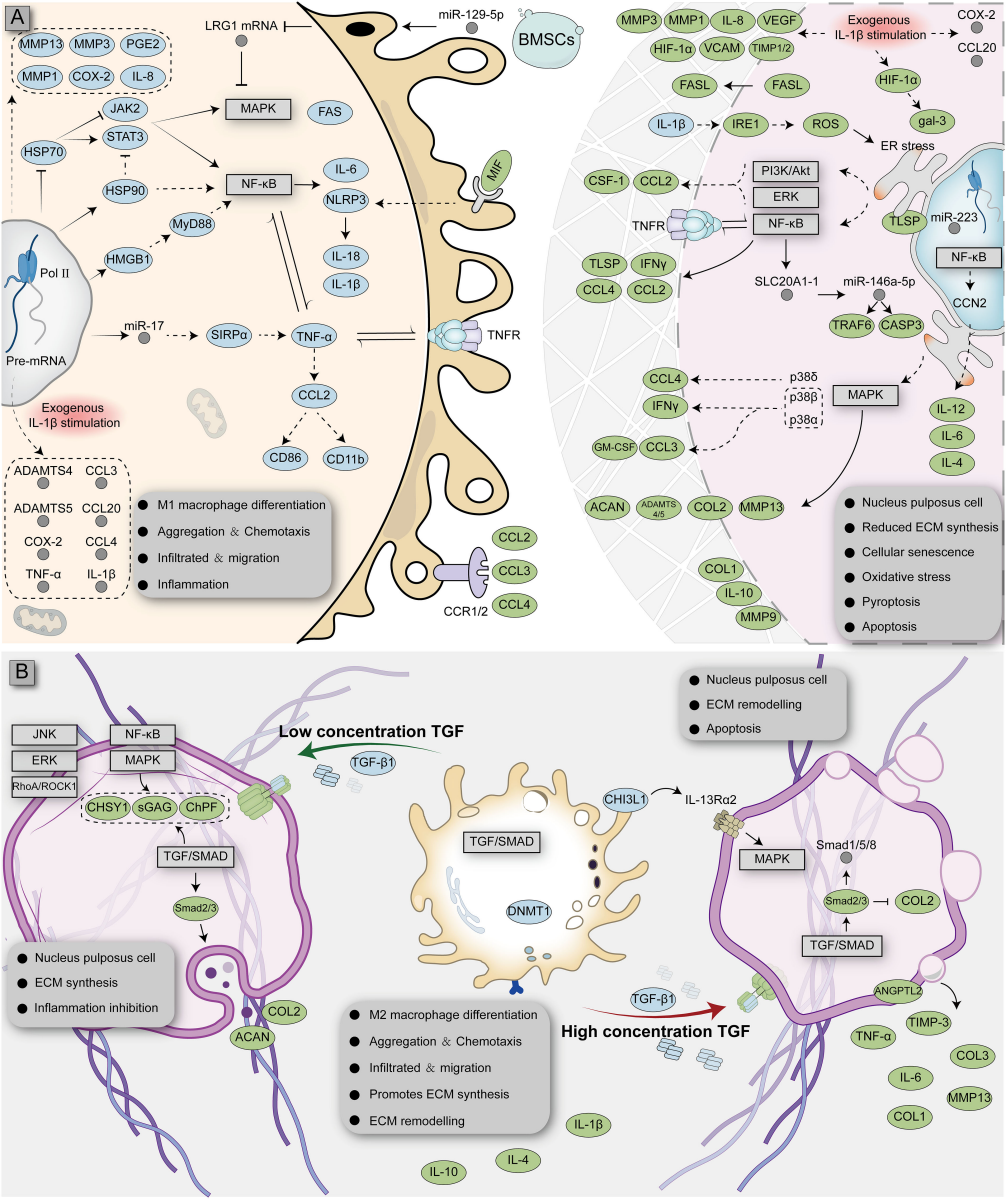
Complex interaction network of macrophages with NPCs affected by different microenvironments. Monocytes differentiate into M1 **(A)** or M2 **(B)** macrophages and interact with NPCs, respectively. The blue ellipse represents the protein produced by macrophages; the green ellipse represents the protein produced by nucleus pulposus cells; the gray circle represents RNA; the rectangular box represents the signaling pathway.

Following M1 macrophage infiltration, NPCs break from the original microenvironment and undergo major metabolic changes and even abnormal death. Yang et al. found that the expression of p38 MAPKα, β, and δ is activated in degenerated NPCs; among them, p38α or p38β can induce the release of GM-CSF and IFNγ and promote M1 polarization of macrophages (54). FasL proteins have been shown to be widespread in immune-privileged organs, such as the eyeball (55, 56). Normal NPCs release FasL protein, which induces apoptosis of immune (macrophages and CD8+ T cells) and endothelial cells, which can attenuate angiogenesis and immune cell recruitment and act as an immune barrier (56–58). However, with changes to the microenvironment, HIF-1α binds to the gal-3 hypoxia response element in NPCs, promoting the expression of gal-3 and reducing the FasL-mediated immune barrier effect (59). HIF-1α expression, in turn, modulates the response of NPCs to oxygen (promoting vascular infiltration and ECM degradation by activating the expression of IL-8, VEGF, MMP-1, and MMP-3 and reducing the expression of VCAM, TIMP-1, and TIMP-2), which leads to further immune exposure (60, 61). Takayama et al. found that degenerated NPCs induce monocyte chemoattractant protein (MCP)-1 and MMP3 expression by activating the MAPK-ERK and PI3K-AKT pathways, leading to the migration of M1 macrophages and disc degeneration (62). A more detailed interaction between M1 macrophages and NPCs is shown in **Figure 2** and **Table 1**.

**Table 1:** Interaction between M1 macrophage and NP.

Study	Species	Experiment	Molecular change	Phenotype change
(37)	Rat	***In vitro***: IL-1β stimulates NPC ***In vivo***: Acupuncture IDD model	**NPC**: ADAMTS5^*^, MMP3/9/13^*^, ACAN^#^, COL1/2^#^, ROS^*^, NF-κB^*^, PI3K/Akt^*^, MAPK^*^ **M1**: IRE1^#^, COX2/5a^*^、CCL2^*^, TNFα^*^ **M2**: CD206^#^	**NPC**: Apoptosis **M1**: Differentiation **M2**: Differentiation inhibition
(40)	Human	***In vitro***: TNFα stimulates NPC **Clinical sample**	**NPC**: SLC20A1-1^*^, miR-146a-5p^#^, TRAF6^*^, Caspase3^*^ **M1**: TNFα^*^	**NP**: Degeneration **NPC**: Apoptosis, growth inhibition **M**: Recruitment, migration
(49)	Human, rat	***In vitro***: Coculture M1 with NPC ***In vivo***: Acupuncture IDD model **Clinical sample**	**NPC**: COL2^#^, ACAN^#^ **M1**: TNF-α^*^, IL-1β^*^, MMP13^*^, NF-κB^*^, MAPK^*^, HSP70^#^, JAK2^*^, STAT3^*^	**NPC**: Inflammation, degeneration **M1**: Differentiation, migration
(63)	Human, mice	***In vitro***: Coculture M1 (RAW264.7) with NPC (Human)	**NPC**: COL2^#^, NO^*^	**NPC**: Cell viability inhibition, oxidative stress **M1**: Differentiation, inflammation
(64)	Human, rat	***In vitro***: Coculture M1 with NPC ***In vivo***: Acupuncture IDD model	**NPC**: ACAN^#^, COL2^#^, MMP13^*^, IL-1β/6/12^*^	**NPC**: Degeneration **ECM**: Catabolism **M1**: Inflammation, differentiation
(48)	Human	***In vitro***: Coculture M1 with NPC	**NPC**: HMGB1^*^, Myd88^*^, NF-κB^*^, NLRP3^*^, Bax^*^, Caspase3/9^*^, MMP3/13^*^, ADAMTS4/5^*^, COL2^#^, ACAN^#^ **M1**: IL-1β^*^, IL-6^*^, TNF-α^*^, IL-18^*^, HMGB1^*^, MyD88^*^, NF-κB^*^, NLRP3^*^	**NPC**: Apoptosis, cell viability inhibition, degeneration **M1**: Differentiation
(51)	Human, mice	***In vitro***: IL-1β stimulates NPC	**NPC**: LRG1^#^, p38MAPK^#^ **M1**: LRG1^#^, p38 MAPK^*^	**NPC**: Apoptosis, degeneration, **M1**: Differentiation
(42)	Human, rabbit	***In vitro***: Coculture M1 (THP-1) with NPC (Human, rabbit) ***In vivo***: Acupuncture IDD model	**NPC**: CCL2/3/5^*^, IL-8^*^, COL2/1^#^, MMP13^*^ **M1**: CCR1^*^, CCR2^*^	**NP**: Degeneration **M1**: Inflammation
(65)	Rat	***In vitro***: Coculture M1 (RAW264.7) with NPC (rat)	**NPC**: Adamts4/5^*^, Mmp3/13^*^, IL-1β^*^, IL-6^*^, Ccl2/3^*^ERK^*^, JNK^*^, Sox9, ACAN^#^, Col2^#^	**NP**: Degeneration **M1**: Inflammation
(66)	Rat, mice	***In vitro***: Coculture M1 (RAW264.7) with NPC (rat)	**NPC**: TNF-α^*^, IL-1β^*^, COX-2^*^, MMP-3/13^*^, ADAMTS-4/5^*^, CCL3/4/20^*^ **M1**: TNF-α^*^, IL-1β^*^, COX-2^*^, MM P-3/13^*^, ADAMTS-4/5^*^, CCL3/4/20^*^	**NP**: Degeneration **M1**: Inflammation **ECM**: Catabolism
(67)	Rat	***In vitro***: Coculture M1 with NPC	**NPC**: TNF-α, IL-1β, MCP-1 **M1**: CD68, TNF-α, MCP-1, MMP, IL-6/8, PGE2, COX2, NO	**M1**: Inflammation
(61)	Human	***In vitro***: IL-1β stimulates NPC and coculture M1 with NPC under hypoxic conditions	**NPC**: IL-6^#^, VCAM^#^, TIMP-1/2^#^, IL-8^*^, VEGF^*^, MMP1/3^*^	**NP**: Degeneration **M1**: Inflammation **ECM**: Catabolism and angiogenesis
(54)	Human	***In vitro***: Coculture M1 with NPC	**NPC**: p38α/p38β MAPK^*^, ADAMTS4/5^*^, MMP13^*^, CCL3^*^, COL2^#^, ACAN^#^	**M1**: Inflammation and differentiate
(55)	Human	***In vitro***: Coculture M1 with NPC	**NPC**: Fasl^*^, disintegrin^*^, AD AM10^*^	**NPC**: Apoptosis **NP**: Degeneration **M1**: Inflammation and migration
(41)	Mice	***In vivo***: Acupuncture IDD model	**NPC**: CCL2^*^ **M1**: TNF-α^*^, CD86^*^, NOS2^*^, YM1^*^ **M2a**: YM1^*^	**M1**: Differentiation, migration, recruitment and inflammation **M2a**: Differentiation and recruitment
(44)	Human	***In vitro*** and ***in vivo***	**NPC**: CCL4^*^, p38-MAPK^*^, NF-κB^*^, TLR-4^*^	**M1**: Migration
(68)	Mice	***In vitro***: LPS stimulates NPC	**NPC**: IL-6/10^*^, GM-CSF^*^	**M1**: Migration and inflammation

*Activate or upregulate; #Downregulate or inhibit; M1, M1 macrophage; M2, M2 macrophage; M, macrophages of unspecified type; NPC, nucleus pulposus cell; NP, nucleus pulposus (nucleus pulposus cell and ECM).

### 3.2 M2 macrophage-NPC crosstalk

The polarization ratio of macrophages is not fixed and is affected by the microenvironment. Macrophages integrate signals from tissues, making them interconvertible between polarization types depending on the amount of cytokines and the duration of immune exposure (69). Whereas M1 macrophage infiltration predominates during the early stages of injury, the number of M2-polarized macrophages near the injured area increases over time as IL-4 (a cytokine that promotes M2-polarization of macrophages) becomes more abundantly expressed (38). Studies have shown that M2 macrophages can inhibit TNF-α-induced apoptosis and senescence of NPCs at the cellular level, which was verified *in vivo* (70). This suggests that M2 macrophages are likely to play a repair-promoting role in the NP. Histopathologically, the typical pathology of M2 macrophage infiltration is high bone turnover and fibrosis, which are inseparable from M2 macrophage function and cytokine release (15). Transforming growth factor (TGF), the most common pro-fibrotic factor released by M2 macrophages, is absent in the normal NP and in the non-granulation area with obvious inflammatory response but is widely distributed across the granulation tissue area rich in M2 macrophages (71). TGF expression was noted to gradually increase with the progressive aggravation of NP degeneration; this not only promoted the polarization of M2 macrophages, but also promoted tissue fibrosis (72, 73). TGF exerts extensive molecular regulatory effects on the NP. Studies have shown that low concentrations of TGF can inhibit CCL3/4 expression and activate Smad2/3, Fas/FasL, RhoA/ROCK, JNK, p38, and ERK1/2MAPK in NPCs, which ultimately promotes the secretion of collagen, proteoglycan, chondroitin polymerizing factor, and sulfated glycosaminoglycans and promotes the inhibition of apoptosis (74–78). However, high doses of TGF can inhibit collagen II but enhance the expression of collagen I/III, TIMP-3, MMP-13, ALK1/5, ANGPTL2, IL-6, TNF-α, and Smad1/2/3/5/8, which induces degeneration of NPCs (79, 80). During communication with NPCs, M2 macrophages can secrete CHI3L1 to activate the IL-13Rα2/MAPK pathway in NPCs to induce ECM metabolic imbalance and NP degeneration (81). IL-10 is also a key anti-inflammatory cytokine released by M2 macrophages, which can inhibit NP degeneration by mediating the p38 MAPK signaling pathway (82).

In general, M1/M2 macrophages signal simultaneously with the NP (NPCs and their ECM), implying the coexistence of damage and repair, although the ratio of damage/repair varies over time (35). When persistent inflammatory stimulation and ineffective healing coexist, the NP interacts with M1/M2 macrophages less intensely, potentially inducing a chronic state, a phenomenon described by Dudli et al. as “frustrated healing response” (15). A more detailed interaction between M2 macrophages and NPCs is shown in **Figure 2** and **Table 2**.

**Table 2:** Interaction between M2 macrophage and NP.

Study	Species	Experiment	Molecular change	Phenotype change
(77, 78)	Human, rat	***In vitro***: TNF-α, TGF-β1 and Fasl stimulates NPC ***In vivo***: Acupuncture IDD model	**NPC**: Caspase3/8^*^, Bcl-2^#^, CCL3/4^*^, Grem1^#^, ERK1/2^#^ **M2**: TGF-β1/smads^*^	**NPC**: pyroptotic inhibition and apoptosis inhibition **NP**: retard inflammation-mediated disc degeneration
(38)	Rat	***In vivo***: Acupuncture IDD model	**M2**: IL-1β^#^, IL-6^*^、TGF-β^*^, IL-4^*^, IL-10^*^	**NP**: Degeneration **M2**: Migration
(70)	Human	***In vitro***: Coculture M2 with NPC ***In vivo***: three-dimensional murine IVD organ culture model	**NPC**: IL-6^#^, MMP-13^#^, ADAMTS-4/5^#^, COL2^*^, ACAN^*^	**NPC**: Proliferation **NP**: Inflammation inhibition, apoptosis inhibition, and senescence inhibition **ECM**: Anabolism
(73)	Mice	***In vivo***: Acupuncture IDD model	**M2**: CD206^*^, TGF-β1^*^, smad2/3^*^	**NPC**: Proliferation **M2**: Differentiation
(64)	Human, rat	***In vitro***: Coculture M2 with NPC ***In vivo***: Acupuncture IDD model	**NPC**: ACAN^*^, COL2^*^, MMP-13^#^, IL-1β/6/12^#^	**NPC**: Proliferation **ECM**: Anabolism **M2**: Differentiation
(81)	Human, rat	***In vitro***: Coculture M2 with NPC	**NPC**: MMP-3/9^*^, COL2^#^, ACAN^#^, ERK^*^, JNK MAPK^*^ **M2a**: CHI3L1^*^ CD206^*^	**NPC**: Degeneration **M2a**: Infiltration
(79, 80)	Human	***In vitro***: High dose TGF-β1 stimulates NPC	**NPC**: COL2^#^, COL1/3^*^, TIMP-3^*^, MMP-13^*^, ALK1/5^*^, ANGPTL2^*^, IL-6^*^, TNF-α^*^, Smad1/2/3/5/8^*^ **M2**: TGF-β1(High dose) ^*^	**NP**: Degeneration
(82)	Rat	***In vitro***: IL-10 stimulates NPC ***In vivo***: Acupuncture IDD model	**NPC**: p38 MAPK^#^, COL2^*^, ACAN^*^, SOX-9^*^	**NP**: retard inflammation-mediated disc degeneration
(83)	Rat	***In vitro***: Coculture M with young or old NPC	**Young NPC**: IL-10^*^, MMP1^#^, IFN-γ^#^ **Old NPC**: IL-10^*^, MMP1^*^, IFN-γ^*^ **M**: NO^*^	Young NP: Higher cell-mediated immunity activity Old NP: Higher humoral immunity activity

*Activate or upregulate; #Downregulate or inhibit; M1, M1 macrophage; M2, M2 macrophage; M, macrophages of unspecified type; NPC, nucleus pulposus cell; NP, nucleus pulposus (nucleus pulposus cell and ECM).

### 3.3 Influence of other factors on macrophage-NPC crosstalk

As mentioned earlier, many syndromes can affect macrophage-NPC crosstalk. High glucose can significantly promote the expression of macrophage markers (F4/80) in mouse NP, accompanied by inflammatory response, increased blood vessel density, and degeneration of collagen fibers (24). A high-glucose environment promotes the accumulation of advanced glycation end products (AGEs) in the intervertebral disc, and the gradually accumulated AGEs can affect the metabolism of the NP through multiple pathways (such as endoplasmic reticulum and mitochondrial homeostasis), which may be closely related to macrophages (84). In a study on obesity and IDD, researchers found that resistin combined with TLR4 increased the expression of CCL4 through the p38-MAPK and NF-κB signaling pathways in NPCs and then induced macrophage infiltration (44). In a study on human clinical NP samples, adipocyte infiltration resulted in increased expression of leptin, adiponectin, and nitric oxide synthase 2 (a marker of pro-inflammatory M1 macrophages) (85); in turn, leptin can regulate the cell cycle of NPCs, activate various signaling pathways (JAK/STAT3, MEK/ERK, PI3K/Akt, and Rho/ROCK/LIMK/cofilin), secrete macrophage chemokines, and induce cytoskeleton reshaping (86). During aging, *Ercc1* ^-^ *^/fl^* mice were bred as a model of immune cell senescence, and degeneration was observed in the intervertebral disc, suggesting a crosstalk between the intervertebral disc and immune cells (6). However, the effects of these syndromes on macrophage-NPC crosstalk are extensive and complex, and current evidence remains limited.

## 4 Macrophage-NP interaction from the perspective of MRI and histopathology

### 4.1 “Black disc” sign, Modic changes, and Schmorl’s nodes

**Figure 3:**
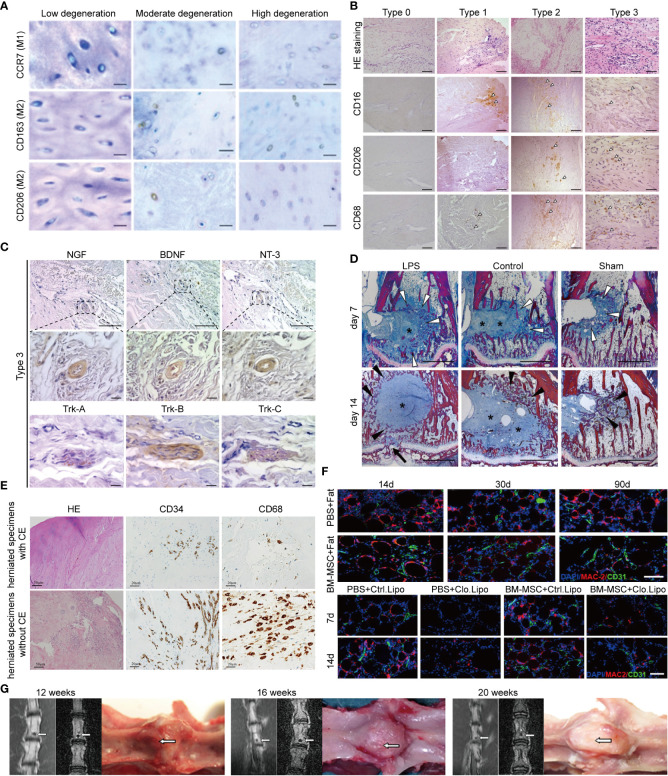
MRI and pathology of immune barrier disruption, “black disk” sign, Modic changes, and Schmorl’s nodes. **(A)** CCR7+, CD163+, and CD206+ macrophages were identified in all moderately and highly degenerated NPs, while few or no positively stained NPCs were found in low-degenerated IVDs, Scale bar = 50 μm. Reprinted with permission form ref (8). copyright (2017) Elsevier. **(B)** Immunohistochemistry showed the distribution of macrophages in the granulation tissue of degenerated NP; type 0, type 1, type 2, and type 3 degeneration was aggravated in turn, and the number of positive cells marked by CD16, CD206, and CD68 gradually increased (arrowheads: positive cells), Scale bar = 50 μm. Reprinted with permission form ref (87). copyright (2021) Elsevier. **(C)** Immunohistochemistry showed nerve growth factor (NGF)-, brain-derived neurotrophic factor (BDNF)-, and neurotrophin-3 (NT-3)-positive vascular endothelial cells in severely degenerated NP granulation tissue; tropomyosin receptor kinase (Trk)-A, Trk-B, and Trk-C in aberrant fiber tissues. Scale bar = 50 or 10 μm. Reprinted with permission form ref (87). copyright (2021) Elsevier. **(D)** Heidenhain-stained sections showed abundant connective tissue within both types of disc surrogates (Figure 5, blue areas around asterisks); at day 14, dense trabecular structures were formed in these areas (black arrow heads); in the LPS group, four of six specimens showed endplate defects at day 14 (Figure 5, black arrow). Reprinted with permission form ref (88). copyright (2017) Elsevier. **(E)** The effect of the presence or absence of CE in hernias on the chemotaxis of macrophages and endothelial cells. Inflamed granulation tissue is observed in herniated specimens without cartilage endplate; CD34-positive capillaries are distributed diffusely in the herniated specimen; CD68-positive macrophages are abundant. CE, Cartilage endplate. Scale bar = 50 or 20 μm. **(F)** Immunofluorescence suggests the tissue remodeling effect of macrophages (angiogenesis and fatty marrow replacement), which may be the key mechanism of Modic type II changes; BM-MSC: Bone marrow mesenchymal stem cell, Clo.Lipo, Clodronate Liposomes (macrophage depleting agent); Scale bar = 50 μm. **(G)** Herniation of autologous NP into the bone marrow, leading to progressive calcification; white arrows indicate the location of herniated NP.

The “black disc” sign indicates the failure of the immune barrier and the establishment of the interaction between macrophages and NPCs. The most typical MRI appearance of NP degeneration is the “black disc” sign. Nakazawa assessed the phenotype and localization of macrophages in an NP with the “black disc” sign. They detected the macrophage markers CCR7, CD163, and CD206 in all degenerated NP (also MRI showed a black disc), and the expression of these markers increased with the grade of degeneration. In contrast, these macrophage markers were barely detectable in the normal NP (non-black discs in MRI) (8) (**Figure 3A**). ECM degradation is considered to be one of the factors directly related to the “black disc” sign (89, 90), and macrophages (mainly the M1 type) widely infiltrate the NP, release a series of cytokines and lysosomal enzymes, trigger inflammation, and change the density of substances in the NP (90). Yamagishi et al. found that in the early stage of immune barrier damage (such as a torn annulus fibrosus), microvessels grow from the damage, accompanied by infiltration of a large number of M1 macrophages, which is manifested as an inflammatory response; in the later stage of immune barrier destruction, M2 macrophages gradually replace M1 macrophages, which manifests as fibrosis and growth of blood vessels and nerves (87) (**Figures 3B, C**).

Modic changes are also a sign of the establishment of interactions between macrophages and NPCs. Some prospective MRI studies have shown that Modic changes significantly accelerate the degeneration of the NP (91, 92). Modic changes themselves occur at the critical sites of the NP immune barrier—the cartilage endplate. However, histological data suggest that Modic changes are involved in the autoimmune response elicited by the NP. Stefan et al. found that persistent inflammatory stimuli during Modic changes originate from the neighboring NP because 1) Modic changes preferentially occur adjacent to the site of disc degeneration; 2) Modic change size is associated with IDD severity; 3) the disc adjacent to Modic releases more pro-inflammatory cytokines; 4) Modic changes generally occur symmetrically proximal and distal to IDD; and 5) Modic changes are coincident with an inflammatory and pro-fibrotic crosstalk with the disc (88). In their subsequent studies, it was proved that Modic changes required the simultaneous presence of cartilage endplate defect and an NP that can amplify the immune response (88) (**Figure 3D**). At the same time, proteomics study of the NP also showed that Modic changes lead to strong inflammatory and host defense responses caused by the crosstalk between the NP and macrophages (93). Embedding autologous NP into lumbar subchondral bone can create Modic animal models, whereas muscle embedding cannot (94). In a herniated granulation tissue (Modic change), there are a large number of CD34-positive capillaries and CD68-positive macrophages, but if the herniated component is mainly cartilage endplate, the infiltration of macrophages is significantly reduced (95–97) (**Figure 3E**). In Modic type II, the expression of M-CSF1, RANKL, RUNX1/2, and other cytokines was significantly increased, and there was infiltration of a large number of M2 macrophages; these M2 macrophages promoted endothelial cell proliferation and differentiation of bone marrow mesenchymal stem cells into adipocytes, whereas macrophage depletion prevented this alteration (96, 98, 99) (**Figure 3F**).

Schmorl’s nodes are generally regarded as focal defects of cartilage endplate and herniation of the NP and lead to calcification and cystic changes, which are similar to Modic type III changes in terms of pathological appearance, location, and related diseases (97). There is already evidence that the immune system, particularly the inflammatory response, plays a key role in Schmorl’s nodes. Embedding rabbit autologous NP into vertebral bodies induced the appearance of Schmorl’s nodes, and the expression of IL-4, IL-17, and IFN-γ was significantly increased, which partly reflects the interaction between NPCs and immune cells (94) (**Figure 3G**). Although this pathological evidence suggests that macrophages may be involved, the specific role of macrophages is still not fully determined.

### 4.2 Rimmed enhancement and NP self-absorption

**Figure 4:**
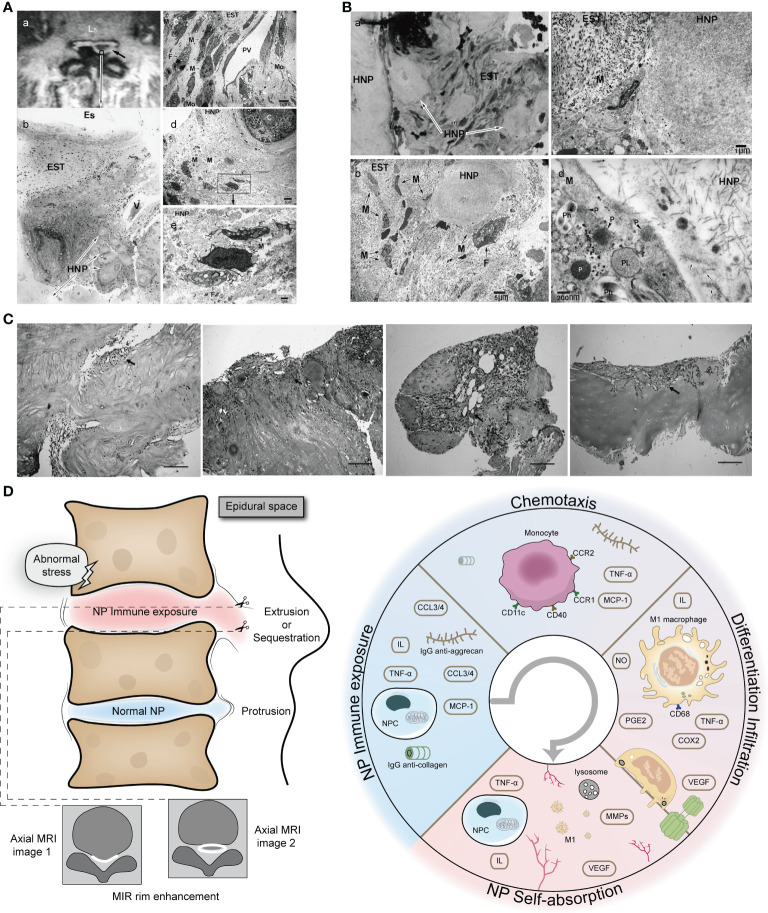
Extensive infiltration of macrophages in herniated NP with rimmed enhancement on MRI. **(A)** Rimmed enhancement MRI image and histopathology of NP. **(A)** herniated NP with rimmed enhancement (black arrow); **(B)** Histological staining (H&E staining) of herniated NP, with obvious inflammatory cell infiltration in epidural scar tissue and NP; **(C)** further electron microscopic examination revealed the following: infiltration of macrophages from the postcapillary venules into the interstitial tissue; numerous macrophages with phagocytic vacuoles and fibroblasts with tubular rough endoplasmic reticulum were present; **(D, E)** electron microscopic examination of the hernia tissue; macrophages phagocytosing interstitial tissue, and fibroblasts with cystic rough endoplasmic reticulum had infiltrated as far as the area around the chondrocytes (Original magnification: b, ×20; c, ×1500; d, ×1200; e, ×5000). Reprinted with permission form ref (4). copyright (2009) Wolters Kluwer. **(B)** Light and electron microscopic examination of the area adjacent to the herniation. Macrophages containing phagosomes and primary lysosomes extensively infiltrated the tissue surrounding the NP (Original magnification: a, ×160; b, ×1200; c, ×5000; d, ×50000). Reprinted with permission form ref (4). copyright (2009) Wolters Kluwer. **(C)** There are new blood vessels and granulation tissue at the edge of the herniated NP. C **(A-D)** With the aggravation of degeneration, the granulation tissue and capillary proliferate massively (black arrow) (H&E, ×100). Reprinted with permission form ref (99). copyright (2013) Springer Nature. **(D)** Schematic diagram of macrophage-NP interaction leading to rimmed enhancement and NP reabsorption. HNP, herniated nucleus pulposus; EST, epidural scar tissue; Es, epidural space; V, venule; F, fibroblast; PV, postcapillary venule; M, macrophage; Mo, monocytes; P, primary lysosome; PL, phagolysosome; Ph, phagosomes.

The occurrence of rimmed enhancement and NP self-absorption indicates a strong M1 macrophage-NP interaction, which also explains why herniated NP with rimmed enhancement is more likely to trigger NP self-absorption. We first describe the general course of the pathology: when the NP is suddenly exposed to the epidural space, the microenvironment of the NP undergoes tremendous changes (from high pressure, hypoxia, and acidic environment to a microenvironment of the epidural space) and causes dramatic alterations in metabolism; the NP exposes the site of autoimmunity and releases a large number of chemokines (such as TNF-α and MCP-1) to recruit monocytes; chemotactic monocytes recognize immune sites in the herniated NP and first polarize into M1 macrophages (decreased expression of the monocyte markers CD11c and CD40 and increased expression of the macrophage CD68 marker), releasing inflammatory factors (such as TNF-α, IL-6, IL-8, PGE2, COX-2, and NO), triggering an inflammatory response and targeting the NP VEGF receptor, inducing vascular infiltration at the edge of the herniation, and promoting granulation formation; the infiltration of microvessels (rimmed enhancement on gadolinium-diethylenetriamine pentaacid MRI) and the release of MCP-1 increase the chemotaxis and accumulation of monocytes and continue to support the mobilization of immune cells to the herniation site; polarized M1 macrophages produce and release a large number of phagosomes and primary lysosomes containing a large number of MMPs, which participate in herniation absorption (4, 36, 65, 67, 99–101) (**Figures 4A-C**).

Not all herniations of the NP are able to initiate self-absorption. The recruitment of M1 macrophages mainly depends on the immunogenicity of the NP because inactivated NP, annulus fibrosus, and cartilage endplate cannot recruit macrophages (36, 101). The more cartilage endplate and annulus fibrosus in a hernia, the more likely they can lead to failure of self-absorption (101, 102). Infiltrated M1 macrophages do not stay in the herniated NP, but further infiltrate from the destroyed area into deep healthy NP and trigger an inflammatory response throughout the NP, thus changing the signal intensity of MRI, which explains why herniated NP is always accompanied by a black disc sign. The role of M2 macrophages in NP self-uptake remains unclear. We speculate that they may play a role in chronic inflammation and repair of damaged immune barrier. A schematic diagram of macrophage-NP interaction leading to rimmed enhancement and NP reabsorption is shown in **Figure 4**.

## 5 Immunological perspectives on the treatment of IDD

### 5.1 Duality of steroid hormones and nonsteroidal anti-inflammatory drugs (NSAIDs) in the macrophage-NP interaction

**Figure 5:**
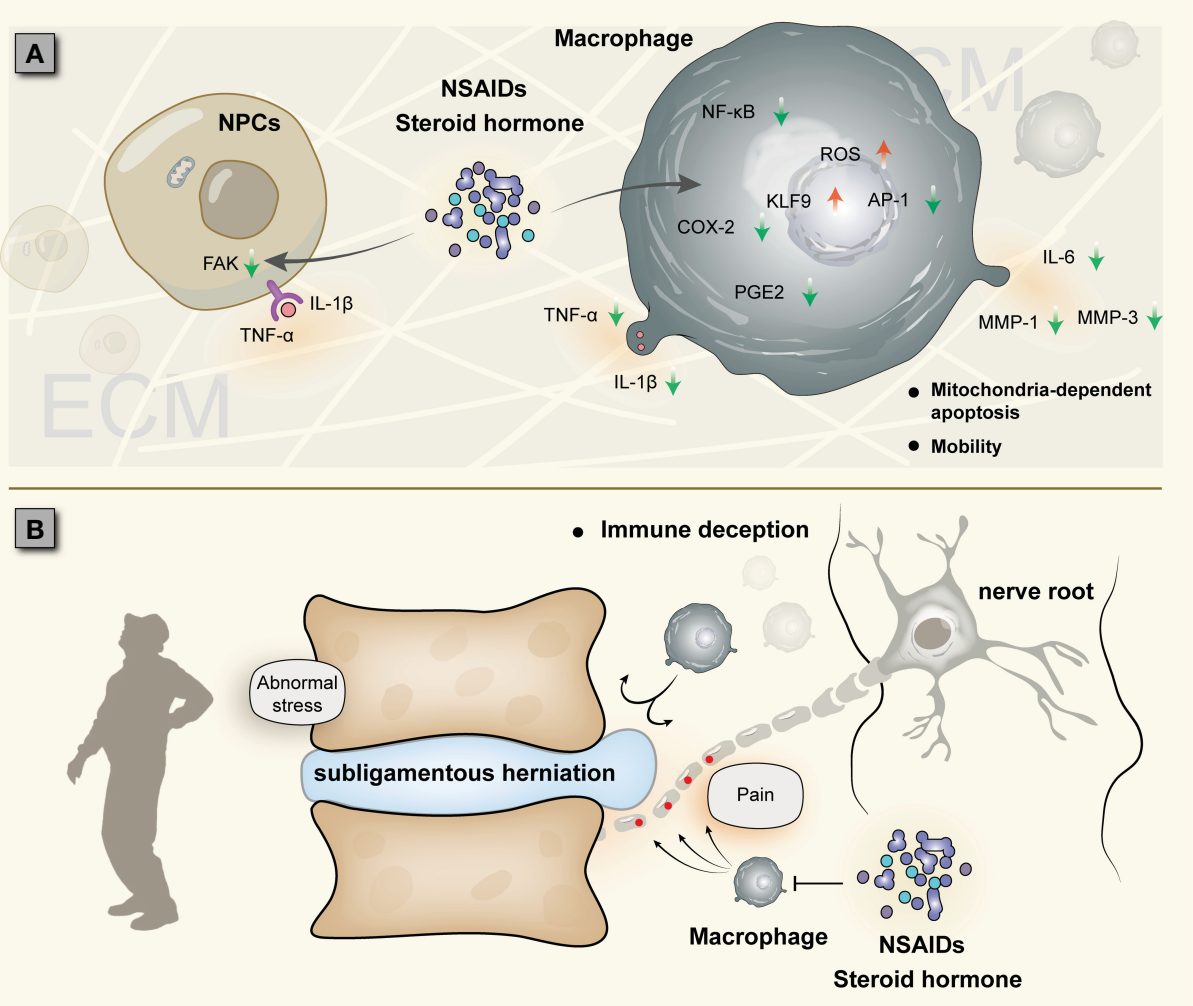
Two cases of IDD treated with steroid hormones and NSAIDs: **(A)** Establishment of macrophage-NP crosstalk and drug suppression of inflammation (possibly converting acute inflammation to chronic inflammation). **(B)** Subligamentous herniation causes nerve injury, and macrophages accumulate around the nerve root instead of the NP; due to the NP immune barrier, macrophage-NP crosstalk is not established, and immune cells cannot phagocytize herniation, which is called immune deception.

Steroid hormones and NSAIDs are often used to treat low back pain and have good short-term anti-inflammatory and analgesic effects (103–105). Studies have shown that dexamethasone induces reactive oxygen species generation and mitochondria-dependent apoptosis in macrophages through KLF9 and inhibits the expression of COX-2, PGE2, and other inflammatory genes downstream of NF-κB (106, 107). Dexamethasone significantly inhibited the release of TNF-α and IL-8 from macrophages (108). NSAIDs also significantly decreased the expression of IL-1β, IL-6, and COX-2 in rat intervertebral discs (109). Teixeira et al. injected diclofenac into bovine intervertebral discs to downregulate the expression of IL-6, IL-8, MMP1, MMP3, and PGE2 (110). In general, macrophages are important targets of steroid hormones and NSAIDs, which can inhibit the release of inflammatory factors, induce abnormal immune function and apoptosis of macrophages, and prevent macrophage-NP crosstalk (106) (**Figure 5A**). However, the reduction or disappearance of pain does not mean that the treatment is successful, especially for acute pain. More and more clinical evaluations have found that the use of steroids and NSAIDs is not ideal for the treatment of acute low back pain (104, 111). Due to NP autoimmunity, acute inflammation aims to rapidly remove foreign bodies recognized by the immune system as a result of the intense interaction of macrophages with the NP, albeit accompanied by pain. The latest research shows that treatment of IDD with steroids or NSAIDs can also lead to prolonged pain, converting acute pain to chronic pain (112).

For NP hernias, the role of steroid hormones and NSAIDs may also be ambivalent. Several studies have shown that epidural steroid injections can reduce inflammation and pain without negatively affecting NP self-resorption (113, 114); other studies, however, have reached inconsistent conclusions that epidural injections are only partially effective and are associated with a very high recurrence rate in IDD (115, 116). The lack of assessment of the integrity of the immune barrier, especially the posterior longitudinal ligament (although challenging by MRI), is one of the reasons for this discrepancy. This is because the perforation status of the posterior longitudinal ligament is closely related to the occurrence of NP self-absorption. A prospective study showed that the self-absorption rate of perforated hernia was 82%, while that of subligamentous hernia was only 48% (117). In the macrophage-NP crosstalk, if the hernia does not break through the immune barrier, it will lead to a large accumulation of macrophages in non-protrusive areas (such as compressed nerve roots), triggering nerve root pain (118). This is essentially neurostimulation induced by mechanical compression, and macrophage-NP interaction is not established. Therefore, no matter how macrophages accumulate, they will not degrade the NP, and the pain cannot be fundamentally relieved, a situation we refer to as “immune deception” (**Figure 5B**). In this case, steroid hormones and NSAIDs are ineffective on macrophage-NP crosstalk. If the immune barrier fails (e.g., in the presence of MRI rimmed enhancement), the use of these drugs can inhibit the inflammatory response of IDD in a short-term, but also inhibit the phagocytosis and degradation of macrophages, which is not conducive to spontaneous remission; at the same time, short-term inhibition does not stop macrophage-NP crosstalk, and IDD will persist. This also seems to explain the high rate of disc herniation recurrence in the long-term follow-up after treatment with these drugs. Inflammation is beneficial to NP self-absorption, but it can also aggravate disc rupture and even cause recurrence of lumbar disc herniation. It is still difficult to find this clinical balance.

### 5.2 Surgical treatment of IDD from the perspective of macrophage-NP interaction

Minimally invasive spine surgical techniques (such as percutaneous transforaminal endoscopic discectomy (PTED) and microendoscopic discectomy) have received increasing attention. However, the problem of postoperative recurrence often challenges the decision of clinicians to use this treatment approach, which limits the development and application of this technology (119). In recent years, researchers have used machine learning to predict the incidence of recurrence and risk factors for recurrence after PTED, including the type and size of intervertebral disc herniation (non-contained herniation), Modic changes, calcification, and obesity (120). As mentioned above, most of these risk factors are related to immune exposure of the NP. Stress changes make the originally abnormal NP more vulnerable to injury after surgery, further aggravating the infiltration of macrophages and exacerbating disc degeneration. Hao et al. found that recurrent disc herniation following percutaneous endoscopic lumbar discectomy preferentially occurs when Modic changes are present (121). Minimally invasive surgery of the intervertebral disc alleviates the mechanical compression of the herniation on the nerve but cannot prevent the crosstalk between immune cells and the NP, which may be an important reason for the recurrence of intervertebral disc herniation. It is worth noting that if it is subligamentous herniation, the posterior longitudinal ligament or annulus fibrosus needs to be removed first during operation, which constitutes artificial destruction of the NP immune barrier. Artificial disruption of this immune barrier may allow for a more adequate interaction between the NP and macrophages, which leads to an increased likelihood of re-herniation. In traditional open surgery, adjacent segmental disc stress is altered, increasing the risk of injury and the possibility of immune exposure of the NP. This may be one of the important causes of adjacent segment degeneration.

### 5.3 Effect of traditional Chinese medicine therapy on macrophage-NP interaction

Acupuncture and traditional Chinese medicine are commonly used for the treatment of low back pain and can regulate the macrophage-NP interaction. Animal experiments have shown that acupuncture can indirectly inhibit IDD and relieve low back pain (122, 123). Acupuncture can regulate the function of immune cells and stimulate macrophages to change from M1 to M2 phenotype; it inhibits the TLR/MyD88, NOD, IκBα/NF-κB, and P38 MAPK pathways in macrophages, subsequently inhibiting the production of inflammasomes and pro-inflammatory mediators; it can also suppress oxidative stress by enhancing superoxide dismutase activity through the Nrf2/HO-1 pathway and eliminate the generation of oxygen-free radicals, thereby preventing macrophage infiltration (124). These inflammatory factors all play an important role in macrophage-NP crosstalk. Many Chinese herbal extracts can suppress inflammation in IDD and may play a role in macrophage-NP interaction. For example, icariin, rhizoma drynariae total flavonoids, and aucubin can inhibit ECM degradation and inflammation (125–127). However, the specific role of these natural herbs in macrophage-NP interaction remains unclear.

## 6 Discussion and perspectives

While the interaction between the NP and macrophages has been studied, the scope and depth for basic biomedical research in this area have been limited. Future research needs to explore in greater detail the origin of autoimmunity of the NP and the molecular mechanisms of macrophage-NP interaction. The mechanisms of IDD are very complex, and macrophage-NP crosstalk is only one of the main types. The currently identified immune cells in IDD include T cells, B cells, and NK cells in addition to macrophages (128). In addition, this review only focused on M1/M2 macrophages and did not include other subpopulations of macrophages, such as CD169 macrophages, T cell receptor macrophages, tumor-associated macrophages (129). Therefore, studies on these cells will make the role of the immune system in IDD more clearer, and immunotherapy of IDD has broad prospects.

## Author contributions

PF was responsible for the collation of data and writing of the original manuscript. YC, CG, and LZ were accountable for the collection of data. JG and NV were responsible for concept development and manuscript revision. All authors contributed to the article and approved the submitted version.
